# An anti-inflammatory and anti-microbial flavone glycoside from flowers of *Cleome viscosa*

**DOI:** 10.1186/2191-2858-2-19

**Published:** 2012-05-21

**Authors:** Musiri Maruthai Senthamilselvi, Devarayan Kesavan, Nagarajan Sulochana

**Affiliations:** 1 Department of Chemistry, National Institute of Technology, Tiruchirappalli, Tamil Nadu 620 015, India

**Keywords:** *Cleome viscosa*, Anti-inflammatory, Anti-microbial, Rat paw edema, Flavonoid glycoside

## Abstract

**Background:**

Natural products isolated from plant sources have been demonstrated as potential candidates against several ailments. The scientific investigations on the underlying principles of phytotherapy can pave way for the convergence of traditional medicines and modern science and technologies.

**Results:**

Quercetin 3-*O*-(2′′-acetyl)-glucoside obtained from ethyl acetate fraction of *Cleome viscosa* is studied against inflammatory of carrageenan-induced rat paw edema ( *in vivo*) and microbial activity on ( *in vitro*). The structure of the glycoside is confirmed by means of hydrogen-1 nuclear magnetic resonance spectroscopy, carbon nuclear magnetic resonance spectroscopy, attached proton test, and mass spectrum. The flavonoid glycoside showed significant anti-inflammatory activity of on carrageenan-induced rat paw edema ( *in vivo*) and anti-microbial activity ( *in vitro*) on *Staphylococcus aureus* (gram positive) and *Escherichia coli* (gram negative). The anti-inflammatory effect of the flavonoid glycoside may be due to the inhibition of prostaglandin synthesis. Selective toxicity with flavonoid glycoside towards the gram-positive bacteria was found on *S. aureus*.

**Conclusions:**

The present study reveals the anti-inflammatory and antimicrobial activities of an isolated quercetin 3-*O*-(2′′-acetyl)-glucoside from a natural source ( *C. viscosa*).

## Background

Natural products isolated from plant sources have been demonstrated as potential candidates against several ailments [1,2]. Our research group has been actively involved in exploring the plant sources for isolation of bioactive compounds as well as their medicinal properties [2-7]. *Cleome viscosa* belongs to the Capparidaceae family. They are distributed in tropical regions. In India, the family is represented by seven genera and about 53 species occurring mostly in the western and southern India. This plant is a common weed found all over the plains of India, with bright yellow flowers. Seeds of this plant are carminative and antiseptic. The juice of the leaves has a pungent flavor.

The methanol extract of *C. viscosa* had been reported to show promising analgesic activity [8], psychopharmacological effects [9], and antipyretic activity [10]. The ethanolic extracts of the leaves, flowers, and roots were tested for antimicrobial activity [11]. The aqueous seed extract [12] as well as ethanolic extract of the leaf powder [13] had showed hepatoprotective activity against carbon tetrachloride-induced liver damage in experimental animal. Crude alcohol and aqueous extracts of the seeds of *C. viscosa* Linn. (Capparidaceae) were investigated for their anthelmintic activity against *Pheretima posthuma*. In the present investigation, the flavonoid glucoside responsible for anti-inflammatory and anti-microbial activity of *C. viscosa* had been isolated and its activities were investigated through *in vivo* and *in vitro* studies.

## Methods

The extraction of the flavonoid glucoside was performed by means of standard fractional distillation method. The detailed experimental procedure is given under experimental section. UV spectra were obtained from on PerkinElmer UV/Vis Spectrometer 301 (PerkinElmer Inc., Waltham, MA, USA). NMR spectra were recorded on Bruker AMX400 Spectrometer (Bruker Corporation, Billerica, MA, USA) at 400.13 MHz for ^1^ H and 75 MHz for ^13^ C using standard Bruker pulse sequences. Tetramethyl silane was used as an internal standard. Mass spectra were recorded in Auto spec FAB^+^ Magnet Bpm: 55 BPI 446544 (AutoSpec Premier, Waters Coporation, MA, USA).

## Results and discussion

The fresh yellow flowers of *C. viscosa* have been found to contain quercetin 3- *O*-(2′′-acetyl)-glucoside. The UV spectrum of the glycoside showed two major absorption peaks at 355 (band I) and 257 nm (band II), showing the presence of a flavonol skeleton. Presence of a 4′-OH group is evident from the bathochromic shift of 48 nm in the NaOMe spectrum of the glycoside. A bathochromic shift of 37 and 77 nm respectively, in AlCl_3_-HCl spectrum and AlCl_3_ spectrum, was the evidence for the presence of a 5-OH group. In the AlCl_3_-HCl spectrum of the glycoside and aglycone, three absorption peaks and a shoulder were seen, which was yet another evidence for the presence of a free 5-OH group. A bathochromic shift of 15 nm in band II observed in the NaOAc spectrum of the glycoside and 16 nm in band II of aglycone indicated the presence of a free 7-OH group. The presence of a 7-OH group was further confirmed by the presence of the shoulder at 327 nm in the NaOMe spectrum of both glycoside and aglycone ring which could be further evidenced from the shift of +21 nm noticed in the glycoside and +17 nm noticed in the case of aglycone in the addition of NaOAc-H_3_BO_3_. In the case of AlCl_3_ spectrum of the glycoside, an absorption peak was present at 432 nm (band I), which upon addition of HCl was reduced by 40 nm. This shows the presence of *O*-dihydroxyl group in the B ring. In the MeOH spectrum of the glycoside, band I was seen at 355 nm and that of the aglycone it was seen at 372 nm, indicating the glycosylation at C-3.

In the hydrogen-1 nuclear magnetic resonance spectroscopy (^1^ H-NMR) spectrum (400 MHz, dimethyl sulfoxide, (DMSO)-*d*_6_, tetramethylsilane, TMS) of the glycoside, the protons at C-6 and C-8 appear as doublets at δ 6.20 and δ 6.40 ppm, respectively. The 5-OH proton appears at δ 12.64 ppm, as a distinct singlet. The C-5′ proton appears as doublet due to ortho-coupling with C-6′ proton at δ 6.86 ppm. The C-2′ and C-6′ protons appear at δ 7.58 ppm. The hydrogen of C-1′ of the sugar moiety is found at δ 5.5 ppm. The remaining glucosyl protons appear in the range of δ 3.0 to 3.5 ppm. The CH_3_ protons of the acetyl group appear as a singlet at δ 1.92 ppm.

The supporting evidence for the structure of the glycoside was provided by the analysis of carbon nuclear magnetic resonance spectroscopy (^13^ C-NMR) (75 MHz, DMSO-*d*_6_, TMS) data. The signal positions and their assignments to the different carbons are banded on the attached proton test spectrum (APT). APT values and their assignments are given in Table 1. From the APT data, numbers of H atoms attached to each carbon atom were determined. -CH_3_ and -CH peaks will be down and -CH_2_ and C with no hydrogen atoms will be up in the APT spectrum [14]. With the help of the APT spectrum, CH_3_, CH_2_, CH, and C with no hydrogen (quaternary carbon atom) were identified in ^13^ C spectra. Due to glycosylation, C-3 carbon shows signal at δ 133.7 ppm and ortho carbon atoms C-2 and C-4 show signals at δ156.0 and 177.1 ppm, respectively. Carbonyl carbon of the acetyl group appears at δ 171.1 ppm. Methyl carbon of the acetyl group appears at δ 21.8 ppm.

**Table 1:** **APT spectral data and their assignments for the glycoside G1 from the flowers of*C. viscosa***

**Chemical shift of carbon atoms (δ, ppm)**	**Type of APT spectra (Up or down)**	**Type of carbon atoms**	**Assignments**
156.0	Up	Quaternary^a^	C-2
133.7	Up	Quaternary	C-3
177.1	Up	Quaternary	C-4
160.9	Up	Quaternary	C-5
98.4	Down	CH	C-6
163.8	Up	Quaternary	C-7
93.3	Down	CH	C-8
156.1	Up	Quaternary	C-9
103.8	Up	Quaternary	C-10
121.3	Up	Quaternary	C-1′
115.0	Down	CH	C-2′
144.5	Up	Quaternary	C-3′
148.2	Up	Quaternary	C-4′
116.3	Down	CH	C-5′
121.32	Down	CH	C-6′
100.1	Down	CH	C-1′′
76.4	Down	CH	C-2′′
73.9	Down	CH	C-3′′
69.9	Down	CH	C-4′′
77.4	Down	CH	C-5′′
61.0	Up	CH_2_	C-6′′
171.1	Up	Quaternary	Carbonyl group
21.8	Down	CH	Methyl group

^a^Quaternary, carbon with no hydrogen.

**Figure 1:**
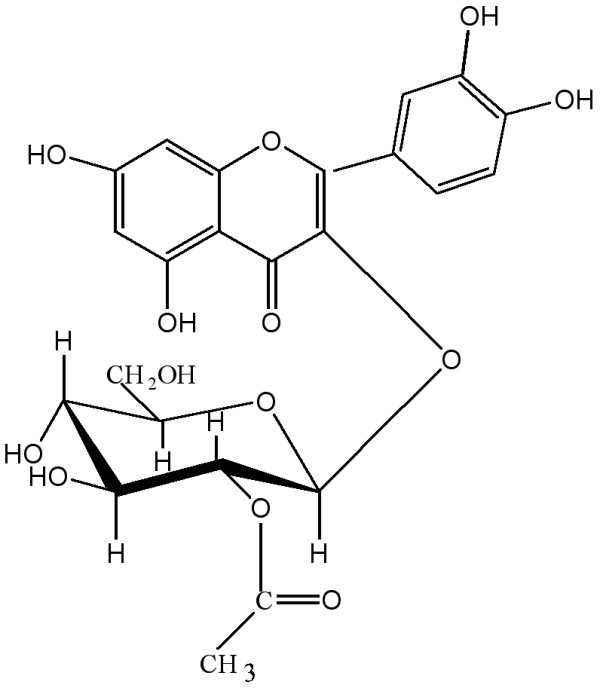
**Quercetin 3-*O*-(2′′-acetyl)-glucoside.** The chemical structure of quercetin 3- *O*-(2′′-acetyl)-glucoside that was isolated from ethyl acetate fraction of *C. viscose.*

The structure of the glycoside was further evidenced by mass spectrum. The spectrum of the aglycone had a peak at m/z 302 for M^+^ ion. The fragmentation pattern following retro-Diels-Alder (RDA), RDA + H and other common fragmentation route [15] is in favor of the identification of the compound. The appearance of a fragment at m/z 137 (RDA) and at 153 (RDA + H) is the evidence for the presence of two hydroxyl groups in ring B and also in ring A. The peak at m/z 165 represented the ion formed from the aglycone through the formation of a five membered ring, which is expected for 3-hydroxy flavones. Peaks at m/z 193 and at m/z 156 are also in favor of the structure of the compound. Based on the above evidences, the glycoside has been characterized at quercetin 3-*O*-(2′′-acetyl)-glucoside (G1) (Figure 1).

Carrageenan-induced inflammation is an acute inflammation. Carrageenan-induced paw edema has been described as biphasic. The initial phase is attributable to the release of histamine, serotonin, and kinin in the first hour after the injection of carrageenan. A more pronounced second phase is related to the release of prostaglandin-like substances in 2 to 3 h. It has been reported that the second phase of edema is sensitive to drugs like phenylbutazone and indomethacin [15]. Table 2 shows that the isolated flavonoid exerted significant anti-inflammatory activity at 100 mg/kg of body weight (BW) during the second phase of inflammation. The significant anti-inflammatory effect of the flavonoid glycoside may be due to the inhibition of prostaglandin synthesis, since it acts in the second phase of inflammation.

**Table 2:** Effect of flavonoid glycoside G1 on carrageenan-induced paw edema

**Drug**	**Dose (mg/kg BW)**	**Paw edema at**		**Increase in paw volume (mL)**	**Percentage of inhibition**
		**(0 h) mL ± SE**	**(3 h) mL ± SE**		
Control	-	0.60 ± 0.01	1.20 ± 0.02	0.60	-
G1	50	0.69 ± 0.01	1.10 ± 0.02	0.41	31.6
	100	0.66 ± 0.01	0.95 ± 0.02	0.29	51.7
	200	0.65 ± 0.01	0.98 ± 0.02	0.33	45.0
Phenylbutazone	100	0.62 ± 0.01	0.80 ± 0.02	0.18	70.0

Gram-negative bacteria *Escherichia coli* has been inhibited to a lesser extent as compared to the gram-positive bacteria *S. aureus* (Table 3). This suggests that there exists a pattern of selective toxicity with the flavonoid glycoside towards the gram-positive bacteria. This conclusion is in agreement with that of the findings of the earlier researchers that flavonoids of the plant origin could selectively inhibit gram-positive bacteria [16]. The glycoside would have interacted with cell wall materials causing their lysis. The integrity of the cytoplasmic membrane might have got damaged causing death of the cell. Alteration of protein and nucleic acid molecule is another possibility. Apart from these things, enzyme action, which is the potential target, can be modified with drugs causing serious repair in the cell.

**Table 3:** Effect of flavonoid glycoside G1 on the growth of bacteria

**Drug**	**Concentration (mg/mL)**	**Zone of inhibition (mm)**		**Inhibition (percent)**	
		***S. aureus***	***E. coli***	***S. aureus***	***E. coli***
Penicillin	1	8	-	100	-
Norfloxacin	1	-	16	-	100
G1	1	5	3	62.50	18.75

## Experimental

### Plant material, extraction, and isolation

The fresh flowers of *C. viscosa* (750 g) collected at Musiri of Tiruchirappalli district, India were extracted with 85% methanol (4 × 500 mL) under reflux. The alcoholic extract was concentrated *in vacuo* and the aqueous extract was fractionated with petroleum ether (60°C–80°C) (3 × 250 mL), peroxide-free Et_2_O (5 × 250 mL) and ethyl acetate (EtOAc) (4 × 500 mL). The petroleum ether fraction did not yield any isolable material.

The Et_2_O fraction was concentrated *in vacuo* and left in the ice chest for a few days. A yellow solid was separated. It came out as yellow needle (m.p. 317°C–318°C) on crystallization from methanol. It was sparingly soluble in hot water and soluble in organic solvents. It gave a golden yellow color with NH_3_ and NaOH and red color with Mg-HCl. It answered Wilson's boric acid test and Molisch's test and responded to Horhammer-Hansel test, Gibb's test, and Wilson's boric acid test. It had ***λ***_max_ MeOH 256, 271, 301sh, 372; +NaOMe 247, 327sh, 413; and + NaOAc 272, 329 nm. It was identified as quercetin.

The residue from EtOAc fraction afforded yellow crystals on crystallization from methanol (m.p. 220°C–221°C). It developed a yellow color when viewed under UV light with and without NH_3_. It developed a green color with alcoholic Fe^3+^ and red color with Mg-HCl. It answered Wilson's boric acid test and Molisch's test but did not answer Horhammer-Hansel test. It had ***λ***_max_ MeOH 257, 299, 355; +NaOMe 263, 327sh, 403; and + NaOAc 272, 320sh, and 354 nm. The ^1^ H, ^13^ C-NMR, and APT spectra were recorded and interpreted.

The glycoside (50 mg) was dissolved in hot aqueous MeOH (5 ml, 50%). An equal volume of H_2_SO_4_ (7%) was added to it. This mixture was refluxed at 100°C for 2 h. The excess of alcohol was distilled off and the resulting solution was extracted with Et_2_O. The residue obtained was studied for the aglycone. It answered Horhammer-Hansel test, Gibb's test and Wilson's boric acid test but did not answer Molisch's test. It had ***λ***_max_ MeOH 253,267,291sh, 335; +NaOMe 266,329sh, 390; and + NaOAc 278, 329sh, 335 nm. A mass spectrum was recorded for aglycone moiety. The filtrate after the aglycone was neutralized with BaCO_3_. The concentrated filtrate was examined through paper chromatography. The identity was confirmed by comparison with an authentic sample of glucose.

### Anti-inflammatory activity

For the investigation of anti-inflammatory activity of the flavonoid glycoside, rat paw edema was used. This method is based on the inhibition of the swelling induced in rat paw. The quantum of the swelling is measured by determining the thickness of the paw, its weight, and the amount of water or mercury it displaces. Healthy albino rats of either sex weighting between 120 and 200 g were selected for the studies. The right paw of each animal was taken as the comparison and left paw for injecting carrageenan. The flavonoid glycosides were dissolved in sterile water to get the desired concentration. The drugs were injected at doses of 50, 100, and 200 mg/kg BW to different groups of animals. Another group of animals received the standard drug, phenylbutazone (100 mg/kg BW), while the other group served as control. A 0.1 mL of 1% solution of carrageenan was injected into the plantar region of the left hind paw of all the animals. The swelling of the paw was measured at different time intervals. The results were expressed as the increase in foot volume in milliliters over the initial volume.

### Antimicrobial activity

In the present study, paper disc agar diffusion method was used to evaluate the antimicrobial activities of the isolated flavonoid glycosides. The bacteria used were *S. aureus* (gram positive) and *E. coli* (gram negative). Nutrient agar was used to cultivate the organism. It comprises of peptone, meat extract, beef extract, and sodium chloride which were properly mixed and heated briefly in the streamer. A 2% agar was added and dissolved by heating. This medium was used for antimicrobial susceptibility testing. Beef infusion, casein acid hydrolysate, starch, and aqueous agar were used. All the above ingredients were mixed in distilled water and dissolved by heating. The mixture was sterilized by autoclaving at 121°C for 15 minutes. The selected stains were subcultured in nutrient broth and these cultivated organisms were used for seeding. The standard drugs used were penicillin and norfloxacin.

The Muller-Hinton agar medium was poured into Petri plates kept on a level surface. The depth of the medium was approximately 4 mm. After the medium got solidified, the plates were allowed to dry for some time by placing them in an incubator about 35°C to 37°C. Pure culture was used for sensitivity testing. Four to five colonies were selected and transferred into a tube containing 5 mL of liquid nutrient medium with the help of a biological loop. The culture was incubated at 35°C to 37°C for 2 to 5 h to obtain moderate turbidity. This was later transferred aseptically into the agar medium and incubated. Filter paper discs (Whatman no.1, Sunrise International (Filter Division), Mumbai, India) with 5.6 mm diameter were punched out. These discs were placed in Petri dishes allowing a distance of 2 to 4 mm between each disc, and the whole was sterilized in a hot air oven at 160°C for 1 h. After allowing the disc to cool, they were impregnated with isolated flavonoid glycoside solution of required concentration. Placing the Petri dishes in a desiccator with lids slightly raised dried the discs. The plate of agar medium was inoculated with the test organism and flavonoid solution. Following incubation, the plates were observed for a zone of inhibition around the drug.

## Conclusions

The isolated flavonoid glycoside (quercetin 3-*O*-(2′′-acetyl)-glucoside) was investigated for its anti-inflammatory activity on carrageenan-induced rat paw edema ( *in vivo*) and antimicrobial activity ( *in vitro*) on *S. aureus* (gram positive) and *E. coli* (gram negative). The significant anti-inflammatory effect of the flavonoid glycoside may be due to the inhibition of prostaglandin synthesis. Selective toxicity with the flavonoid glycoside towards the gram-positive bacteria was found on *S. aureus*.

## Abbreviations

APT: Attached proton test; BW: Body weight.

## Competing interests

The authors declare that they have no competing interests.

## Authors’ information

MMS is a professor and head of Department of Chemistry, EVR College Tiruchirappalli, India. Her doctoral work at National Institute of Technology-Tiruchirappalli (NIT-T) was mainly focused on isolation of natural products and their biomedical applications. She is actively working in natural products and green chemistry. DK is a research assistant at Institute of High Polymers Research, Shinshu University, Japan. His master's work at National Institute of Technology, Tiruchirappalli (NIT-T) was mainly focused on electrochemistry of synthetic and natural organic compounds. He is actively working on biopolymers. NS is a professor of the Department of Chemistry at NIT-Tiruchirappalli (on contract). NS is a renowned researcher in the field of phytochemistry/medicinal chemistry and has published more than 130 articles in peer-reviewed international and national journals. Her field of expertise ranges from natural products to electro-organic chemistry.
